# Rabies in a Dog Imported from Egypt — Connecticut, 2017

**DOI:** 10.15585/mmwr.mm6750a3

**Published:** 2018-12-21

**Authors:** Yonette Hercules, Nelva J. Bryant, Ryan M. Wallace, Randall Nelson, Gabriel Palumbo, Jemeila N. Williams, J. Miguel Ocana, Sheryl Shapiro, Hilaire Leavitt, Sally Slavinsk, Alexandra Newman, David A. Crum, Brian E. Joseph, Lillian A. Orciari, Yu Li, Pamela Yager, Rene E. Condori, Kendra E. Stauffer, Clive Brown

**Affiliations:** ^1^Division of Global Migration and Quarantine, National Center for Emerging and Zoonotic Infectious Diseases, CDC; ^2^Division of High-Consequence Pathogens and Pathology, National Center for Emerging and Zoonotic Infectious Diseases, CDC; ^3^Connecticut Department of Public Health; ^4^New York City Department of Health and Mental Hygiene; ^5^New York State Department of Health; ^6^Maryland Department of Health; ^7^Washington State Department of Agriculture.

## Abstract

In 2007, the United States successfully eliminated canine rabies virus variant. Globally, however, dogs remain the principal source of human rabies infections. Since 2007, three cases of canine rabies virus variant were reported in dogs imported into the United States, one each from India (2007), Iraq (2008), and Egypt (2015) (*1*–*3*). On December 20, 2017, a dog imported into the United States from Egypt was identified with rabies, representing the second case from Egypt in 3 years. An Egyptian-based animal rescue organization delivered four dogs from Cairo, Egypt, to a flight parent (a person solicited through social media, often not affiliated with the rescue organization, and usually compensated with an airline ticket), who transported the dogs to the United States. The flight parent arrived at John F. Kennedy International Airport (JFK) in New York City and, via transporters (persons who shuttle dogs from one state to another), transferred the dogs to foster families; the dogs ultimately were adopted in three states. The Connecticut Department of Public Health Laboratory (CDPHL) confirmed the presence of a canine rabies virus variant in one of the dogs, a male aged 6 months that was adopted by a Connecticut family. An investigation revealed the possibility of falsified rabies vaccination documentation presented on entry at JFK, allowing the unvaccinated dog entry to the United States. This report highlights the continuing risk posed by the importation of dogs inadequately vaccinated against rabies from high-risk countries and the difficulties in verifying any imported dog’s health status and rabies vaccination history.

## Case Report and Findings

On December 20, 2017, a shipment of four rescue dogs arrived at JFK from Cairo, Egypt. Two transporters and one owner retrieved the dogs, with planned distribution to foster homes and permanent owners in Connecticut, Maryland, and Virginia. A fifth dog on the flight, traveling with a separate flight parent and not part of this shipment, shared the cargo hold and was temporarily housed in New Jersey and West Virginia before reaching its Washington destination. One of the four dogs, a male Chihuahua mix aged 6 months (dog A), was noticeably agitated and bit the flight parent before boarding the plane in Egypt. Dog A was imported with tooth fractures and exposed maxillary bone, reportedly from being struck by a car in autumn 2017.

On assessment at a Connecticut veterinary clinic on December 21, dog A exhibited hyperesthesia (increased sensitivity to stimuli) and paresis. The dog bit a veterinary technician during a blood draw procedure and died shortly thereafter. The clinic submitted brain tissue for rabies testing to CDPHL. On December 26, CDPHL confirmed rabies virus infection by direct fluorescent antibody testing and informed CDC. On December 28, CDC confirmed the direct fluorescent antibody results and determined the variant was consistent with Africa 4 subspecies canine rabies virus circulating in Egypt (Figure).

**FIGURE:**
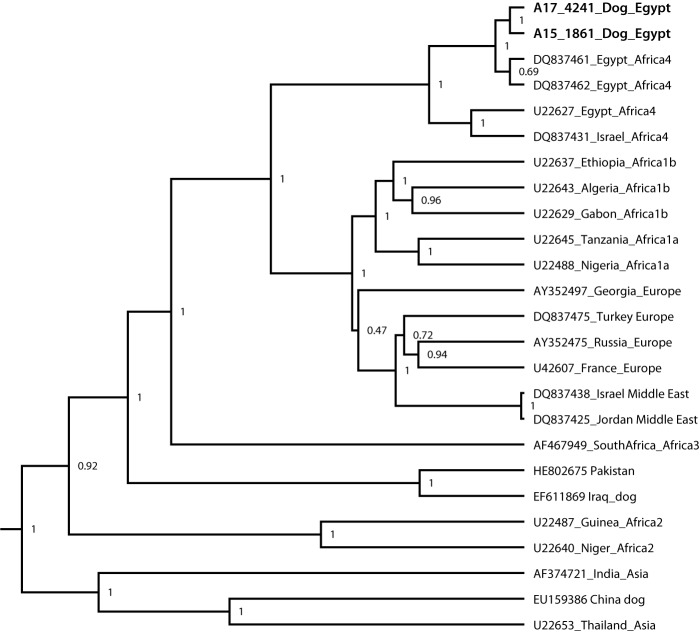
Egyptian dog (bolded for both 2017 and 2015 isolates groups) with other available Egyptian strains as Africa 4 subspecies canine rabies virus (RABV Africa 4) subspecies* * Phylogenetic tree is constructed from 1350 nucleotides of nucleoprotein gene using BEAST program (http://beast.community). Posterior probabilities were labeled at each branch with probability values between 0 and 1. Branch length is related to the number of nucleotide substitutions. The more substitutions, the longer the branch. More evolved strains will be further from their ancestor.

## Public Health Investigation

After CDPHL’s notification of confirmed rabies, CDC’s New York Quarantine Station initiated a contact investigation to identify animals or persons potentially exposed to dog A during its infectious period (10 days before symptom onset until death [December 9–21]). CDC contacted health departments in the chain of distribution of all five dogs in the cargo hold to initiate rabies exposure assessments; these health departments included the Maryland Department of Health, Virginia Department of Health, New York City Department of Health and Mental Hygiene, New York State Department of Health and Mental Hygiene, and Washington State Department of Health. The investigation also included U.S. Customs and Border Protection (CBP), the U.S. Department of Agriculture’s Animal and Plant Health Inspection Service, the airline that transported the animals, and the domestic cargo offloading company at JFK.

State health department staff members interviewed dog A’s caretakers, volunteers, and employees associated with the involved rescue groups and veterinary hospital staff members for potential exposure. Public health investigators for Maryland, New Jersey, New York, Washington, and West Virginia determined that the animal transporters and foster home volunteers had no direct contact with dog A; therefore, no postexposure prophylaxis (PEP) was recommended for those persons. Connecticut public health officials, in accordance with national guidelines (*4*), recommended PEP for the flight parent bitten in Cairo, the caretakers of dog A, and the veterinary technician who was bitten. CDC and CBP conducted a contact investigation to identify potentially exposed persons and animals at JFK. CBP interviewed the airline’s U.S.-based cargo staff members and reviewed surveillance video to identify transporters and CBP staff members who had potential exposure to dog A. CBP identified 13 cargo and baggage handlers and four CBP officers; New York City Department of Health and Mental Hygiene conducted risk assessments and determined that PEP was not recommended. All handlers reportedly wore gloves while handing the crates and had no direct contact with the dogs. CBP reviewed the importation paperwork and cleared the animals but had no physical contact with the dogs or the crates.

The domestic animal exposure investigations determined that all four dogs in the Egyptian shipment (dogs A, B, C, and D) were individually crated within the airplane cargo hold. A fifth dog (dog E, also in an individual crate), that was not part of the rescue organization shipment, shared the same cargo hold space. The animals were never removed from the crates during shipment, so they could not have had direct contact with dog A. Therefore, dogs B, C, D, and E were not considered exposed to dog A during transport. Dog A had no contact with any dogs after exiting the airport and was placed in isolation at the veterinary clinic. All five dogs had certificates indicating rabies vaccination both at ≥3 months and ≥30 days before arrival at a U.S. port of entry (Table), as required by CDC dog importation regulations (*5*). However, because dog A’s infection raised uncertainty about the validity of rabies vaccination for the five dogs, investigators determined that the four remaining dogs from the shipment should receive a rabies booster vaccination followed by confinement, as recommended by the Compendium of Animal Rabies Prevention and Control (*6*). In light of this uncertainty and the potential for unreported exposure before shipment, Maryland Department of Health elected to confine dogs B and C for 4 months; Virginia Department of Health and Washington State Department of Health elected to confine dogs D and E for 30 days (Table). Egyptian public health investigators instituted vaccination, confinement, and monitoring for four other dogs in the Egyptian rescuer’s possession and indicated that persons exposed to dog A were given PEP. Clarification by Egyptian authorities of why an appropriately vaccinated dog (according to the documentation provided) developed rabies is pending.

**TABLE:** Date or year of birth and reported rabies vaccination or revaccination dates for five dogs shipped from Egypt to the United States on December 20, 2017

Dog	Information provided on Egyptian rabies vaccination certificate		Vaccination or revaccination after arrival in the United States		
	Date or year of birth	Date of rabies vaccination	Final U.S. destination	Date of U.S. rabies vaccination or revaccination	End (duration) of confinement*
A	Jun 10, 2017	Sep 14, 2017	Connecticut	N/A	N/A
B	2013	Nov 22, 2017	Maryland	Jan 5, 2018	May 5, 2018 (4 months)
C	Jun 9, 2017	Nov 2, 2017	Maryland	Dec 26, 2017	Apr 26, 2018 (4 months)
D	2012	Oct 27, 2017	Virginia	Dec 27, 2017	Jan 26, 2018 (30 days)
E	Apr 6, 2016	Nov 4, 2017	Washington	Dec 28, 2017	Jan 27, 2018 (30 days)

**Abbreviation: **N/A = not available.

* Includes CDC-required confinement period of 30 days after vaccination and individual state requirements for rabies postexposure quarantine.

## Discussion

Elimination of the canine rabies virus variant from the United States required approximately 5 decades and hundreds of millions of dollars. Imported cases present an ongoing opportunity for reestablishment of the variant and require lengthy and costly investigations to prevent additional cases in both humans and animals.

This report describes the sixth importation of a rabid dog into the United States in the past 15 years and the third from the Middle East; all six were rescued dogs (*1*–*3*,*7*,*8*). Rabies in dogs might be underreported in the United States because rabies can have a variable clinical course that might not prompt animal owners to seek postmortem rabies testing (*9*). Previous reports and publications have discussed the public health challenges associated with the global movement of animals in commerce and the federal, state, and local authorities involved with dog importation (*1*–*3*,*7*,*8*). The United States has one of the most robust rabies surveillance and response networks in the world, with approximately 120 diagnostic laboratories testing approximately 100,000 animals every year. This network of clinical veterinarians, public health practitioners, and rabies diagnostic laboratories improves the chances of early detection of cases and termination of transmission chains. A high level of background vaccination in most U.S. dog populations also serves as a barrier to this disease. This surveillance network rapidly identified these six documented events, and none has resulted in transmission in U.S. dogs.

CDC and local and state agencies have received reports of invalid or questionable health and rabies vaccination certificates for imported dogs (*9*). The inadequacy of dog A’s rabies vaccination could have been caused by vaccination failure, improperly stored vaccine, or fraudulent documentation. Vaccination failure is rare when rabies vaccine is properly stored and administered; no other vaccination issues were reported from the manufacturer with the lot used in dog A. In addition, dog A was apparently not part of the original shipment agreed to by the flight parent, who had no medical history for dog A. Accepting rescue dogs or other animals without knowing their histories or having personal knowledge about the accuracy of veterinary documents can lead to the unnecessary exposure of persons and animals to a lethal zoonotic disease.

To prevent the reintroduction of the canine rabies virus variant, the United States needs to continue vigilance at ports of entry, domestic surveillance infrastructure, and dog vaccination coverage. At U.S. ports of entry, there is a visual inspection for death or signs of illness that prompts a required necropsy or veterinary examination under CDC’s regulations. However, the signs typical of rabies (e.g., agitation, barking, aggressiveness, and altered mental status) also are common in stressed dogs during long-distance travel, and, unless the animal is near death, ill dogs could be overlooked. Increased education of rescue organizations both domestically and internationally and enhanced focus on dogs from countries where canine rabies virus variant is circulating could help increase awareness of the significance of rabies control in dog importations and reduce the potential for importation of cases.

**Summary**

**What is already known about this topic?**

Public health challenges associated with the global movement of animals include importation of canine rabies virus variant into the United States from countries where the virus is enzootic.

**What is added by this report?**

A rabid dog imported into the United States from Egypt, with documentation of rabies vaccination but no medical history, resulted in a six-state investigation and administration of rabies postexposure prophylaxis to multiple persons.

**What are the implications for public health practice?**

Use of flight parents who have no medical history for the dog they are transporting poses a potential human and animal health threat. To prevent reintroduction of the canine rabies virus variant, the United States needs to continue vigilance at ports of entry, domestic surveillance infrastructure, and high dog vaccination coverage.
